# Measuring provider well-being: initial validation of a brief engagement survey

**DOI:** 10.1186/s12913-023-09449-w

**Published:** 2023-05-03

**Authors:** Megan Call, Fares Qeadan, Benjamin Tingey, Ellen Morrow, David Webber, Blake Hamilton, Amy Locke

**Affiliations:** 1 Department of Psychiatry, University of Utah Health, 501 Chipeta Way, UT 84108 Salt Lake City, USA; 2 Resiliency Center, University of Utah Health, 26 S 2000 E, Salt Lake City, UT 5775A84112 USA; 3 Parkinson School of Health Sciences and Public Health, Loyola University Chicago, 2160 S. First Avenue, Maywood, IL 60153 USA; 4 Department of Surgery, University of Utah Health, 30 N 1900 E, Salt Lake City, UT 84132 USA; 5 University of Utah Health, Medical Group, 50 N. Medical Drive, Salt Lake City, UT 84132 USA; 6 Department of Family & Preventive Medicine, University of Utah Health, 375 Chipeta Way Ste A, UT 84108 Salt Lake City, USA

**Keywords:** Brief assessment, Professional well-being, Burnout

## Abstract

**Background:**

Measurement is one of the critical ingredients to addressing the well-being of health care professionals. However, administering an organization-wide well-being survey can be challenging due to constraints like survey fatigue, financial limitations, and other system priorities. One way to address these issues is to embed well-being items into already existing assessment tools that are administered on a regular basis, such as an employee engagement survey. The objective of this study was to assess the utility of a brief engagement survey, that included a small subset of well-being items, among health care providers working in an academic medical center.

**Methods:**

In this cross-sectional study, health care providers, including physicians and advanced clinical practitioners, employed at an academic medical center completed a brief, digital engagement survey consisting of 11 quantitative items and 1 qualitative item administered by Dialogue™. The emphasis of this study was on the quantitative responses. Item responses were compared by sex and degree, domains were identified via exploratory factor analysis (EFA), and internal consistency of item responses was assessed via McDonald’s omega. Sample burnout was compared against national burnout.

**Results:**

Of the 791 respondents, 158 (20.0%) were Advanced Practice Clinicians (APCs), and 633 (80.0%) were Medical Doctors (MDs). The engagement survey, with 11 items, had a high internal consistency with an omega ranging from 0.80–0.93 and was shown, via EFA, to have three domains including communication, well-being, and engagement. Significant differences for some of the 11 items, by sex and degree, in the odds of their agreement responses were found. In this study, 31.5% reported experiencing burnout, which was significantly lower than the national average of 38.2%.

**Conclusion:**

Our findings indicate initial reliability, validity, and utility of a brief, digital engagement survey among health care professionals. This may be particularly useful for medical groups or health care organizations who are unable to administer their own discrete well-being survey to employees.

**Supplementary Information:**

The online version contains supplementary material available at 10.1186/s12913-023-09449-w.

## Background

The association between the well-being of health care professionals (HCPs) and metrics related to quality, safety and the overall performance of health care systems is well-documented [1]. Burnout among HCPs is strongly correlated with lower patient satisfaction and treatment compliance, and increased rates of medical error, hospital infections, malpractice litigation, work-related interpersonal conflict, and staff turnover [2–6]. HCPs experiencing burnout are more likely to be dissatisfied with their work and are at an increased risk for mood and anxiety disorders, substance misuse and substance use disorders, and suicide [7–12]. This dire combination of system and individual level consequences stresses the critical importance of assessing the well-being of HCPs, ideally on a regular basis and along with other standard practices of institutional performance measurement. Understanding how well-being influences other institutional performance metrics, such as financial performance and patient satisfaction, can facilitate the implementation of better tailored and more effective improvement interventions that will have a sustainable and lasting impact on the health care system.

There are many ways to measure the well-being of HCPs across an organization. Commonly used instruments range from comprehensive assessment to single-item burnout measures. In a National Academy of Medicine discussion paper, Dyrbye and colleagues highlight common considerations for system-wide well-being measurement and specifically recommend that the data being collected is important to stakeholders, is widely applicable and actionable, can detect and reflect changes in the institution, causes limited burden to respondents and the organization, and is founded on items that exhibit a sufficient level of construct validity [13]. These insights and availability of multiple assessment options have helped further well-being measurement across health care systems; however, survey administration remains challenging due to coordination of other surveys, potential survey fatigue and low response rates, limited finances and time and personnel for data collection and interpretation, or the perceived need to focus on other matters.

One way to address these competing issues and conundrums is to include well-being measures on employee engagement surveys, which are administered across health care systems on a consistent basis. Previous research indicates that incorporating well-being items into employee engagement surveys creates a more informative data set and adds richness to data interpretation, such as understanding how leadership communication influences employee burnout and satisfaction or analyzing how the combination of employee resilience and burnout influences patient experience [14, 15]. More needs to be done to understand the different ways well-being and engagement can be measured and the how the data collected can be interpreted and acted upon. The purpose of this study was to evaluate the utility of an emerging, brief, digital engagement survey that includes a small subset of well-being items. Our efforts were guided by the following three research questions: 1) What is the internal consistency of item responses? 2) What is the construct validity of the assessment instrument? 3) Do respondents answer the engagement survey differently based on provider role?

## Methods

### Participants

University of Utah Health (U of U Health) annually surveys all academic faculty and staff employed within the health sciences campus to measure employee engagement. This survey is administered by Dialogue™, formerly known as Waggl™, which is a digital, organizational engagement survey company [16]. U of U Health contracts with Dialogue™ for administration of the survey, use of their questionnaire items, access to their reporting software, and utilization of the population bank of their qualitative assessment responses. The present cross-sectional study focuses on the 791 physicians and advance practice clinicians who completed the survey either in January or April 2019. Participation was voluntary, and all data were confidential. Although Dialogue™ tracks responses by employee identification number, identifying information was not available to any U of U Health employee. The ethical approval and informed consent to participate was waived by the U of U Health Internal Review Board (IRB# 00,124,369). All methods were carried out in accordance with relevant guidelines and regulations.

### Measurement

The engagement survey consisted of 11 quantitative items and 1 qualitative item to measure employee satisfaction, opportunities for professional development and advancement, job-related resources, workplace communication, and well-being. The complete list of items is in supplemental Table 1. Eight of the survey items were from the Dialogue™ question bank. These items were derived from the field of employee engagement research and selected through expert consensus. They have been used broadly throughout the healthcare industry [17]. The remaining three items, specifically those measuring work control, workplace stress and burnout, were adapted and modified from the Mini-Z worklife survey in order to fit the direction of the agreement scale of the instrument. The Mini-Z survey has demonstrated moderate reliability, with Cronbach’s alpha of 0.8 for the complete measure, and good internal validity [18]. Additionally, the single-item measuring burnout is highly correlated with the emotional exhaustion scale of the Maslach Burnout Inventory [19]. The 5-point Likert scale of the Dialogue™ instrument had the following anchors: 1 = strongly disagree, 2 = disagree, 3 = neutral, 4 = agree and 5 = strongly agree. Responses for each quantitative item were dichotomized by agreement with “strongly agree” and “agree” being recategorized into a “Yes” response and “neutral,” “disagree” and “strongly disagree” being recategorized into a “No” response. Participants who responded to the quantitative items were included in all analyses, regardless if they completed the survey in its entirety.

**Table 1:** Characteristics of participants (overall and stratified by degree)

	**Overall**	**APC**	**MD**	***P*-value**^**c**^
	n (%^a^)	n (%^a^)	n (%^a^)	
**Total**	791^b^	158	633	
**Age 30–49** n (%)				0.44
No	281 (35.5)	52 (32.9)	229 (36.2)	
Yes	510 (64.5)	106 (67.1)	404 (63.8)	
**Gender** n (%)				< 0.001
Female	356 (45.4)	118 (77.6)	238 (37.6)	
Male	429 (54.7)	34 (22.4)	395 (62.4)	
**Race Ethnicity** n (%)				0.51^d^
White	619 (78.3)	132 (83.5)	487 (76.9)	
Asian	55 (7.0)	8 ( 5.1)	47 ( 7.4)	
Hispanic	14 (1.8)	2 ( 1.3)	12 ( 1.9)	
Other	13 (1.6)	1 ( 0.6)	12 ( 1.9)	
Prefer not to say	74 (9.4)	14 ( 8.9)	60 ( 9.5)	
Everyone else	16 (2.0)	1 ( 0.6)	15 ( 2.4)	
**Appointment Type** n (%)				< 0.001
Mostly Clinical	709 (89.6)	155 (98.1)	554 (87.5)	
Mostly Other	36 (4.6)	3 ( 1.9)	33 ( 5.2)	
Mostly Research	46 (5.8)	0 ( 0.0)	46 ( 7.3)	
**Degree** n (%)				-
APC	158 (20.0)	-	-	
MD	633 (80.0)	-	-	

^a^column %’s;

^b^One physician removed, counts may not add up to total due to removal of missing rows;

^c^Chi-Square test (unless otherwise noted);

^d^Fisher’s Exact test

Demographic information was collected via population from human resources records when participants completed the survey. Available demographic variables included age, sex, race and ethnicity, faculty appointment type (research or clinical), and academic degree. Data from the sole qualitative item, “What would make you feel more appreciated at work? How would this be impactful?” is not included in the present study.

### Statistical analyses

Descriptive demographic information was summarized overall with counts and percentages because all variables were categorical. With provider type (i.e., physician vs advance practice clinician) being the primary exposure variable of interest, demographics were also stratified by this variable and characteristic comparisons between physicians and advance practice clinicians were made using a Chi-Square test for sufficiently large sample sizes, and Fisher’s Exact test for small sample sizes. For analysis of the 11-dichotimzed items, item agreement percentages were presented overall and stratified by demographics of interest (i.e., sex and degree status) while being compared with a Chi-Square test. Additionally, odds ratios with 95% confidence intervals (CIs) were presented to assess the odds of agreement for each item between the stratified groups.

While individual items alone provided useful insights, of greater interest were underlying trends seen across combinations of these items. As such, an exploratory factor analysis (EFA), with iterated principal axis factor extraction and squared multiple correlations on the diagonal of the correlation matrix, was conducted on the 11 quantitative items on their original scale. This was done to confirm the validity of grouping certain items into domains. Orthogonal varimax and oblique promax rotations were examined, and upon findings of high correlations between factors as well as findings yielding more of a simple structure (see Supplemental Figs. 1a-1b) the promax rotation was used for final results. Rotated factor pattern loadings (correlations between items and factors) were provided to determine the item domains. Item communalities (proportion of variance of each item contributed by the factors) as well as inter-factor correlations were also provided. Diagnostics were assessed to confirm an optimal number of factors and overall factor solution. As a sensitivity analysis, the EFA was repeated while changing the extraction method to maximum likelihood and minimum residual to confirm stable loadings of the domains.

To measure the internal consistency of item responses, McDonald’s omega was used [20]. This was preferred over the commonly used Cronbach’s alpha due to its ability to handle multiple latent dimensions in the item responses (as opposed to one item-wide dimension “unidimensionality” for alpha), correlation between errors (alpha assumes independence between errors), violation of tau-equivalence (different factor loadings of the items while alpha assumes all are equal), and the overall outperformance of omega over alpha under such situations [21–29]. Thus, with factors underlying the items, as well as correlations between those factors, total omega and hierarchical omega were employed to consider these phenomena. In addition, the algebraic greatest lower bound (GLBa) was used as a companion, which has been shown to be a reliable estimate in the presence of non-normal or skewed data [28, 30–33].

An additional analysis consisted of comparing the sample percentage of burnout (those who answered “strongly disagree” or “disagree” to the item “Burnout is not a problem for me”) to the national percentage using a one-sample z hypothesis test for proportions and a 5% significance level.

As a sub-analysis, domains from the EFA were converted into weighted factor scores. Because domains were shown to be correlated, all demographic predictors were fit simultaneously in multivariate linear regression with all domains as outcomes. Thus, models were fit each with a different outcome and involving all the same predictors. Coefficients, however, across all models covaried. To confirm selection of predictors, a multivariate analysis of variance (MANOVA) Pillai test was conducted to determine which predictors were jointly contributing to all outcomes significantly. Adjusted beta-hats ($\widehat{\beta }$_ADJ_) were calculated for predictors, which reported the average change in domain outcome with each one-unit increase in predictor. Significance of predictors was reported with *p*-values. Model diagnostics were assessed for predictor/outcome sets to ensure optimal fit. To capture uncertainty of estimates, while owing to the fact multiple sets of coefficients were present that covaried, 95% confidence ellipses were plotted to capture uncertainty in two dimensions (two outcomes were plotted at a time, and all combinations were assessed). The ellipse captured the area within which one could be 95% confident that the true joint domain outcome was contained. With the predictors of gender and degree being of interest, all comparisons considered these predictors while holding all others constant at mean levels.

All other hypothesis tests (besides the one sample z-test) were two-sided with a significance level of 5%. All analyses were performed in SAS, version 9.4 (SAS Institute Inc).

## Results

### Sample characteristics

Of the 1,447 providers invited to participate in the survey, 791 completed the survey (54.7%) and had responses eligible for analysis. The total number of respondents accurately reflected the demographics of providers within U of U Health regarding sex, race/ethnicity, age, professional degree, and role within the institution. More specifically, the total U of U Health provider population consisted of higher percentages of males (51.8%), between the ages of 30–49 years old (68.1%), white race (78.3%), physicians (83.0%) and primarily clinical workers (80.4%). Survey respondents consisted of higher percentages of males (54.7%), between the ages of 30–49 years old (64.5%), white race (78.3%), physicians (80.0%) and primarily clinical workers (89.6%; Table 1). Compared to APCs, physicians had significantly higher percentages of males (62.4% vs. 22.4%, P < 0.001) and lower percentages of mostly clinical work yet higher percentages of mostly research work (clinical: 87.5% vs. 98.1%; research: 7.3% vs. 0.0% *P* < 0.001). No significant differences were found in age or race/ethnicity between APCs and physicians (*P* = 0.44 and 0.51 respectively; Table 1). These comparisons were similar to comparisons between the total population of APCs and physicians who worked at U of U Health. More specifically, total U of U Health APCs had a higher percentage of females (77.0%) in comparison to U of U Health physicians (43.0%). APCs and physicians were both primarily white race (84% vs 76.9%) and between the ages of 30–49 years old (75.6% vs 67.9%). These demographic comparisons are also similar to comparisons between the population of APCs and physicians working in the state of Utah [34–36].

### Item comparisons by sex and degree

The item agreement responses are displayed in Tables 2 and 3. Overall, as much as 95% agreed to the statement “I am motivated to do my best” while as low as 41% agreed to the statement “Burnout is not a problem for me.” Compared to females, males reported significantly higher agreement percentages to the statements “I have adequate opportunities to advance my career at U of U Health” (71.1% vs. 61.2%; *P* = 0.004), “I have control over workload” (50.8% vs. 37.6%; *P* < 0.001), “My work-related stress is manageable” (73.9% vs. 61.2%; *P* < 0.001), and “Burnout is not a problem for me” (48.7% vs. 31.2%; *P* < 0.001) (Table 2).

**Table 2:** Agreement responses by sex

Items^a^	Overall	Female	Male	*p*-value ^c^	OR^d^ (95% CI)
	n (%^b^)	n (%^b^)	n (%^b^)		
**Total**	791^e^	356^e^	429^e^		
I would recommend UUH	693 (87.6)	320 (90.0)	371 (86.5)	0.14	0.72 (0.46, 1.12)
I see myself working at UUH	668 (84.5)	305 (85.7)	360 (83.9)	0.50	0.87 (0.59, 1.29)
I am motivated to do best	754 (95.3)	343 (96.4)	406 (94.6)	0.25	0.67 (0.33, 1.34)
I have adequate opportunities	523 (66.1)	218 (61.2)	305 (71.1)	0.004	1.56 (1.16, 2.10)
My supervisor keeps me informed	545 (68.9)	237 (66.6)	307 (71.6)	0.13	1.26 (0.83, 1.71)
I can express opinions	568 (71.8)	253 (71.1)	313 (73.0)	0.56	1.10 (0.80, 1.50)
My input is sought	500 (63.2)	216 (61.0)	283 (66.0)	0.12	1.26 (0.84, 1.68)
I have access to tools	584 (73.8)	258 (72.5)	324 (75.5)	0.33	1.17 (0.85, 1.61)
I have control over workload	352 (44.5)	134 (37.6)	218 (50.8)	< 0.001	1.71 (1.29, 2.28)
My worked-related stress is manageable	537 (67.9)	218 (61.2)	317 (73.9)	< 0.001	1.79 (1.32, 2.43)
Burnout is not a problem for me	322 (40.7)	111 (31.2)	209 (48.7)	< 0.001	2.10 (1.56, 2.81)

^a^Items dichotomized to “Yes” with responses: “strongly agree”, “agree”; “No” with responses: “neutral”, “disagree”, “strongly disagree”;

^b^Column %’s;

^c^Chi-Square test;

^d^odds ratio (comparing male to female);

^e^counts may not add up to total due to removal of missing values

**Table 3:** Agreement responses by degree

Items^a^	Overall	APC	MD	*p*-value ^c^	OR^d^ (95% CI)
	n (%^b^)	n (%^b^)	n (%^b^)		
**Total**	791	158	633		
I would recommend UUH	693 (87.6)	135 (85.4)	558 (88.2)	0.36	1.27 (0.77, 2.10)
I see myself working at UUH	668 (84.5)	132 (83.5)	536 (84.7)	0.73	1.09 (0.68, 1.75)
I am motivated to do best	754 (95.3)	150 (94.9)	604 (95.4)	0.80	1.11 (0.50, 2.48)
I have adequate opportunities	523 (66.1)	69 (43.7)	454 (71.7)	< 0.001	3.27 (2.29, 4.68)
My supervisor keeps me informed	545 (68.9)	99 (62.7)	446 (70.5)	0.06	1.42 (0.99, 2.05)
I can express opinions	568 (71.8)	113 (71.5)	455 (71.9)	0.93	1.02 (0.69, 1.50)
My input is sought	500 (63.2)	85 (53.8)	415 (65.6)	0.01	1.64 (1.15, 2.33)
I have access to tools	584 (73.8)	117 (74.1)	467 (73.8)	0.94	0.99 (0.66, 1.47)
I have control over workload	352 (44.5)	47 (29.8)	305 (48.2)	< 0.001	2.20 (1.51, 3.19)
My worked-related stress is manageable	537 (67.9)	94 (59.5)	443 (70.0)	0.01	1.59 (1.11, 2.28)
Burnout is not a problem for me	322 (40.7)	54 (34.2)	268 (42.3)	0.06	1.41 (0.98, 2.04)

^a^Items dichotomized to “Yes” with responses: “strongly agree”, “agree”; “No” with responses: “neutral”, “disagree”, “strongly disagree”;

^b^Column %’s;

^c^Chi-Square test;

^d^odds ratio (comparing MD to APC)

By degree, physicians also reported a significantly higher agreement percentage than APCs to the statement “I have adequate opportunities to advance my career” (71.7% vs. 43.7%; *P* < 0.001). Whereas males were 1.56 times more likely to report agreement to this statement than females, physicians were 3.27 times more likely to report agreement than APCs. Again, similar to sex, physicians reported significantly higher percentages of agreement to the items of “workload control”, “manageable work-related stress”, and “lack of burnout” (burnout p-value on the boundary of significance: 0.06). Physicians also reported 65.6% agreement to the statement “My input is sought and considered” whereas APCs only reported 53.8% (*P* = 0.01; Table 3).

### Exploratory factor analysis

**Fig. 1:**
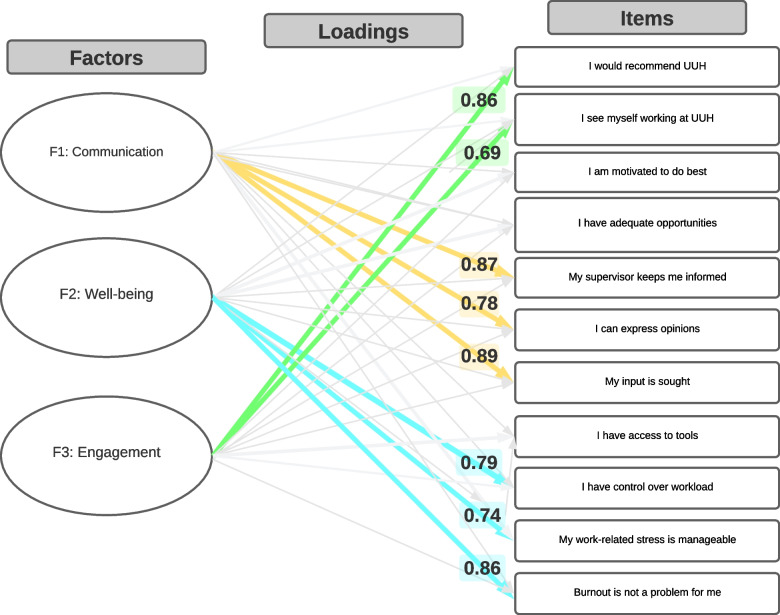
Rotated factor pattern loadings. (Iterated principal axis factor extraction with promax rotation)

A final count of three factors was determined by various methods including content expertise on the number of expected domains, a scree plot (Supplemental Fig. 2) with an elbow in eigenvalues at three factors, discontinuation of high loadings beyond three factors, and negligible change of root mean square of the residuals (RMSR) beyond three factors (RMSR = 0.02). Finally, the Tucker Lewis Index of factoring reliability was 0.979 again indicating an optimal factor solution. Tables 4, 5 and Fig. 1 show the results of the EFA. The factors were identified by the highest loadings and were characterized as: (1) communication (“My supervisor keeps me informed,” “I can express opinions without fear of retribution,” “My input is sought, heard, and considered”) (2) well-being (“I have control over my workload”, “My work-related stress is manageable”, “burnout is not a problem for me”) (3) engagement (“I would recommend UUH as a great place to work”, “I see myself working at UUH in two years”). These three factors accounted for 59% of the variability in all the survey items. In addition, the factors exhibited significantly positive correlations with each other. Communication had a correlation of 0.63 with well-being, and a correlation of 0.77 with engagement. Well-being had a correlation of 0.70 with engagement. All correlation significance tests revealed *P* < 0.001. Sensitivity analyses revealed that the loading patterns remained consistent across all extraction adjustments to the EFA (Supplemental Figs. 3, 4 and Supplemental Tables 1a-b, 2a-b). The responses indicated a high internal consistency with a total omega of 0.93, hierarchical omega of 0.80, and GLBa of 0.94.

**Table 4:** Rotated factor pattern loadings^a^

Items	Factor 1	Factor 2	Factor 3	Communality
	**Communication**	**Well-being**	**Engagement**	
***I would recommend UUH***	0.038414	-0.09561	***0.862244***	0.684683
***I see myself working at UUH***	0.041175	-0.06107	***0.693371***	0.467562
I am motivated to do best	-0.04216	0.135854	0.4283	0.2504
I have adequate opportunities	0.483406	0.052128	0.257335	0.545236
***My supervisor keeps me informed***	***0.871667***	0.005525	-0.07559	0.669309
***I can express opinions***	***0.785126***	0.001219	0.045529	0.67496
***My input is sought***	***0.88556***	-0.02692	0.01836	0.779672
I have access to tools	0.199592	0.231791	0.370418	0.52386
***I have control over workload***	0.021167	***0.786908***	-0.05178	0.584344
***My worked-related stress is manageable***	0.049427	***0.743792***	0.04901	0.659345
***Burnout is not a problem for me***	-0.05195	***0.861351***	-0.03516	0.649762

^a^Iterated principal axis factor extraction with promax rotation

**Table 5:** Inter-factor correlations^1^

Items	Factor 1	Factor 2	Factor 3
Factor 1	1.00	-	-
Factor 2	0.63	1.00	-
Factor 3	0.77	0.70	1.00

^1^ From iterated principal axis factor extraction with promax rotation; Pearson’s product-moment correlation significance test revealed p < 0.001 for all bivariate correlation comparisons

### Assessing the state of burnout

The sample percentage of burnout in our study, 31.5%, was significantly lower than the national percentage of 38.2% (221/579), taken from National Data for 579 Clinicians in the ACLGIM Worklife and Wellness Project [17] (*P* < 0.001; Table 6).

**Table 6:** Burnout proportion (*n* = 791)

	**n (%**^**b**^**)**	***P*-value**^**c**^
**Burnout**^**a**^ n (%)		< 0.001
No	542 (68.5)	
Yes	249 (31.5)	

^a^Responses of “Yes” on “Burnout is not a problem for me” taken to be “No” and responses of “No” were taken to be “Yes”;

^b^Column %’s;

^c^One-sample z-test for proportions comparing sample proportion of burnout (31.5%) to national burnout average of 38.2% (221/579), taken from National Data for 579 Clinicians in the ACLGIM Worklife and Wellness Project [17]

### Sub-analysis: multivariate linear regression on factor domains

The MANOVA Pillai test indicated the utility of including all predictors as joint predictors in multivariate modeling (all *P* < 0.001 or on the boundary of significance [i.e. 0.05 < *P* < 0.10]). Compared to those aged 30–39, all other ages had lower outcomes of communication, well-being, and engagement on average. Males had higher outcomes than females, and all other races (compared to white) had lower outcomes. Those doing mostly research, or other, had higher outcomes than those primarily focused on clinical work. Those who were an MD reported higher outcomes than those were APC (Supplemental Table 3). Supplemental Fig. 5a-c show gender/degree predictions for joint outcomes as well as 95% confidence ellipses. When considering outcomes jointly, and across all gender and degree comparisons, all outcomes were positively correlated (increases in one outcome were associated with increases in the other outcomes). Females and APCs had the lowest outcomes whereas males and MDs had the highest outcomes.

## Discussion

These findings demonstrate the initial internal-consistency reliability (via omega), construct validity (via EFA) and utility of a brief engagement survey among HCPs. Three primary domains were identified within the measurement tool: engagement, communication and well-being. While these factors do not encompass an exhaustive assessment of provider well-being, they do provide guidance and consideration for future organizational measurement and research. For instance, the identified well-being domain consists of three items that were developed and adapted to fit the direction of the Likert scale of the brief instrument. This was originally viewed as a potential downside of using this survey tool. Yet, the burnout rate generated from one of these new items was consistent with a previous assessment of burnout among our providers, where we used a measure with well-established reliability and validity, and it was similar to the current national provider burnout rate [18, 37]. This finding contributes to the national conversation of how burnout may be able to be inquired about and measured in a variety of ways, which gives more permission for innovation and flexibility when including well-being items in system-wide assessment.

The remaining two domains also reinforced previous trends related to HCP well-being. Regarding engagement, respondents reported being motivated to do their best almost every day despite approximately one-third of the sample endorsing struggling with burnout and another one-third reporting a neutral response toward experiencing burnout. This observation highlights how the internal drive of HCPs to provide excellent care for patients and be successful at work remains present even during significant exhaustion and possible despair. This finding also calls forth a cruel and costly irony: HCPs experiencing burnout still present to work motivated to do their best despite being at increased risk for causing patient harm [6]. Further understanding of this phenomenon is critical in creating a culture of medicine that supports self-care, boundary setting, and a sustainable, healthy work environment [38]. In addition, the identified communication domain may have implications for understanding psychological safety, an emerging important construct in understanding and addressing group dynamics in healthcare [15, 39].

Between group comparisons with demographics also generated notable results. The similarities and discrepancies in responses found between physicians and APCs were consistent with previous comparisons between these groups [40–42]. Engagement and burnout rates tend to be similar between these roles; however, there is currently more understanding of, and research conducted on, physician burnout. The APCs in our study endorsed higher work-related stress and lower work-related control, less opportunities for career advancement, and a lower sense that their input is sought, heard, and considered in comparison to their physician counterparts. This combination of high stress in conjunction with multiple perceived limitations could have the potential for many APCs to feel trapped in their profession. These findings highlight the need for further investigation on the specific needs of APCs, how they compare to other HCP roles, and how to address these needs in different healthcare settings.

There were three primary differences between male and female participants in our study. Male providers reported higher perceived well-being, work-related control, and opportunities for career advancement in comparison to female providers. These findings are consistent with previous research on gender discrepancies between HCPs [43, 44]. Female physicians are more likely to experience underrepresentation in leadership positions, slower academic promotion, fewer professional awards, fewer opportunities to present at grand rounds or national lectures, and an increased likelihood of harassment, impostor syndrome, and burnout in comparison to male physicians [45]. Our results highlight a continued need to better understand how the risk and protective factors for burnout may differ between male and female providers and how interventions such as established career pipeline programs for burgeoning leaders, effective mentoring programs for female providers, and addressing implicit bias in the workplace may reduce these disparities.

There are limitations to our study. The results of this research are difficult to generalize due to the study sample being from one institution and having a moderate response rate. The study design was cross-sectional, which implies that no causality can be contributed to any identified relationships within our findings. The demographic information in this study was collected via already populated human resources records. It is possible that this information was inaccurate depending on how participants completed the demographic section of the human resources paperwork when they applied for work at our institution. This limited our ability to make between group comparisons, especially among racial/ethnic groups. The brief nature of this survey may have been convenient; however, it was not exhaustive. Other drivers of burnout, such as the impact of the electronic medical record, workplace efficiency, staffing, salary, and support following a workplace trauma, were not measured [46]. This limitation creates an incomplete picture regarding workplace well-being at our institution. In addition, the items utilized in this survey have limited validity. The Dialogue™ items have solely exhibited face validity and the well-being items used in this assessment were not previously validated. It is important to note that some of the Dialogue™ items can read as double-barreled and need to be further reviewed and analyzed for clarity and usability. As far as measures of internal consistency, there was a disparity between the total omega (0.93) and hierarchical omega (0.80). Total omega focuses on information across all factors without specifying the specific variance contributions of sub-factors, while hierarchical omega does take these specific sub-contributions into account [29]. Although the appropriate cutoff for optimal internal consistency can be debated and should depend as well on content expertise, all our reported percentages were no lower than 0.80 and generally coincide with high internal consistency.

## Conclusions

Assessing HCP well-being is an important aspect of acting on the quadruple aim in healthcare settings. Findings from this study suggest opportunities for next steps including further assessing the utility and validity of the burnout item of this measure, further examining the communication domain as a simple way to measure psychological safety in the workplace, and continued investigation of how well-being measurement can be incorporated into already existing organizational assessment practices, like engagement and climate surveys. Understanding how well-being influences other institutional performance metrics, such as financial performance and patient satisfaction, can facilitate the implementation of better tailored and more effective improvement interventions that will have a sustainable and lasting impact on the health care system.

## Supplementary Information

**Additional file 1.** 

## Data Availability

The datasets generated and/ or analyzed during the current study are not publicly available due to 1) the sensitivity of the data (e.g., individual information on provider well-being) and 2) license restrictions from Dialogue™. The data are available from the corresponding author upon reasonable request and with permission of Dialogue™.

## Abbreviations

APC: Advanced practice clinician
CI: Confidence interval
HCP: Health care professional
