# Supplementary material for: Measuring provider well-being: initial validation of a brief engagement survey
Source: BMC Health Serv Res. 2023 May 3;23:432. doi: 10.1186/s12913-023-09449-w (PMC10157943; doi:10.1186/s12913-023-09449-w)
Supplement: Supplementary file 1 — Additional file 1. [file 12913_2023_9449_MOESM1_ESM.docx]

**Supplement**


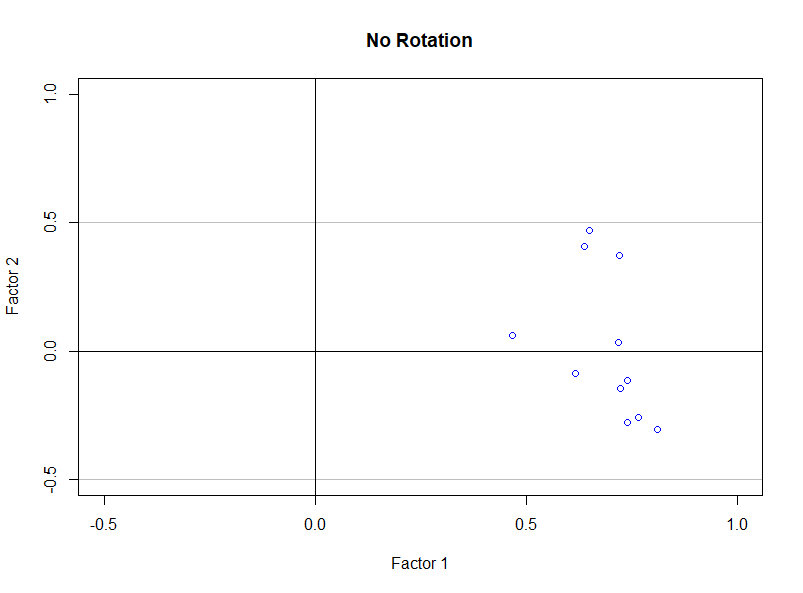

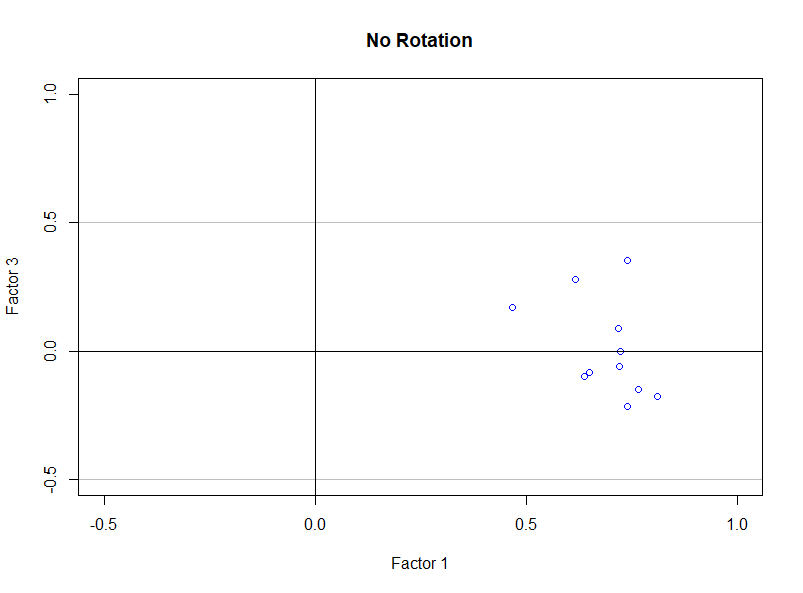


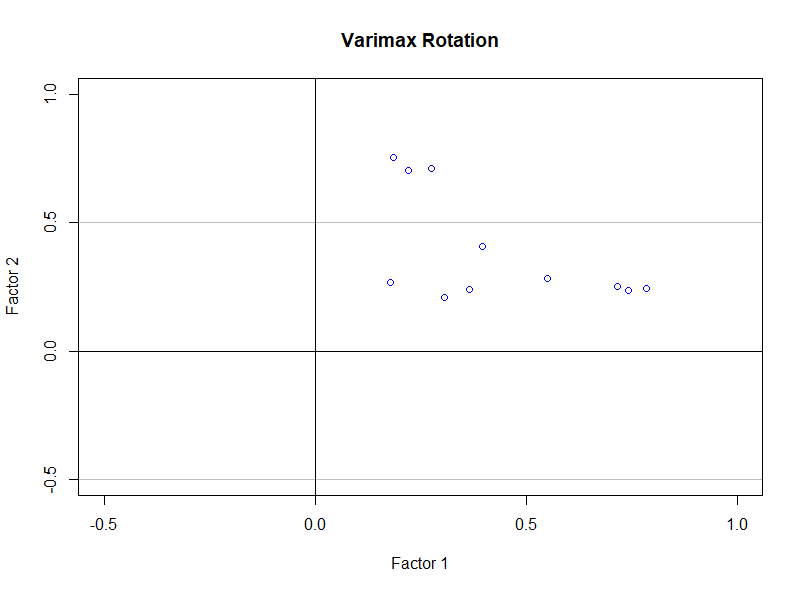

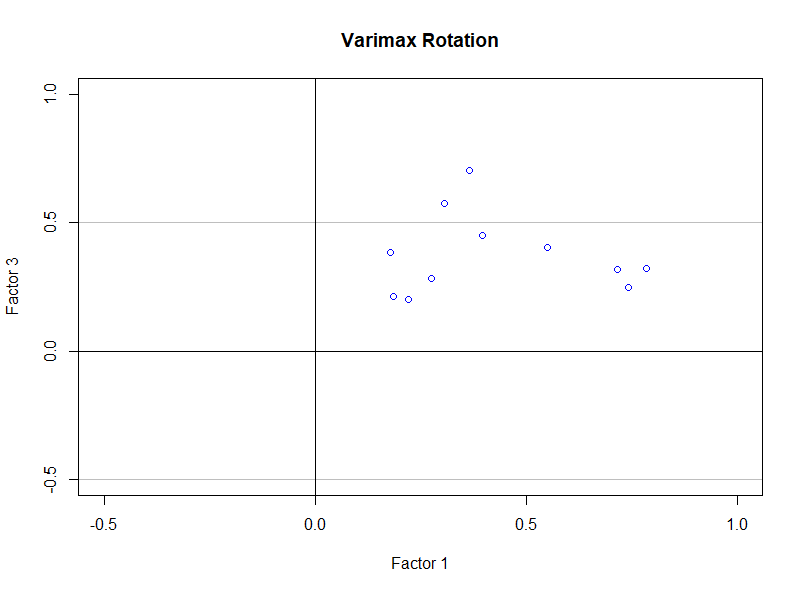


**Figure S1a: Factor pattern loadings under no rotation and varimax rotation**

Under no rotation, item loadings are clustering together whereas under varimax rotation, items are becoming more uniquely focused on one factor


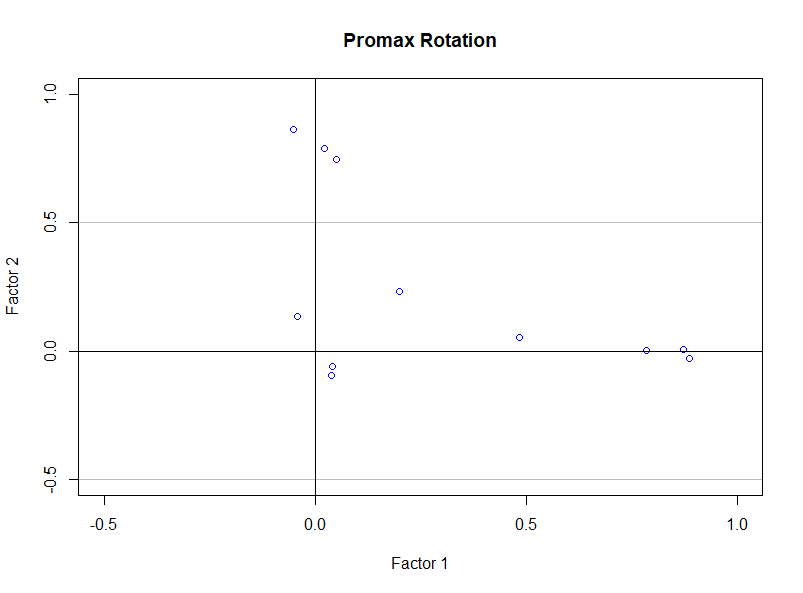

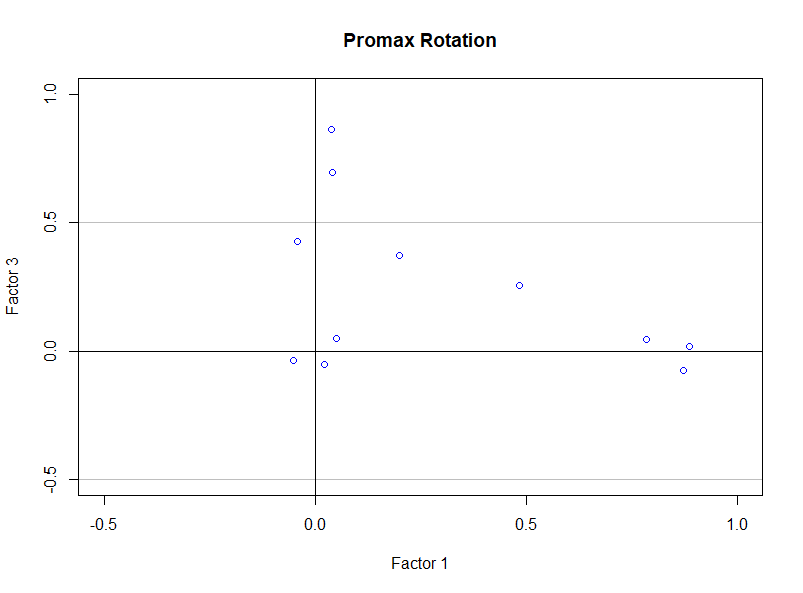


**Figure S1b: Factor pattern loadings under promax rotation**

Under promax rotation, items are becoming even more uniquely focused on one factor


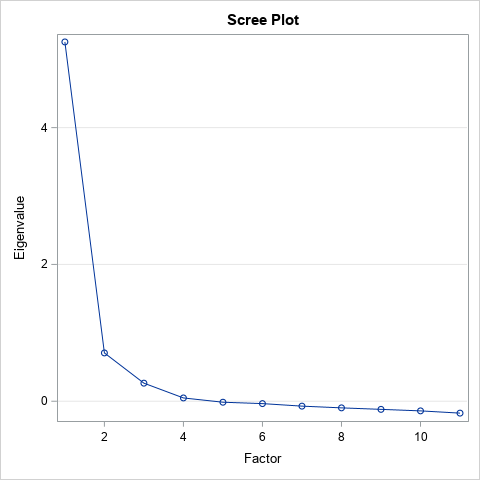


**Figure S2. Scree plot for number of factors selection, iterated principal axis factor extraction**


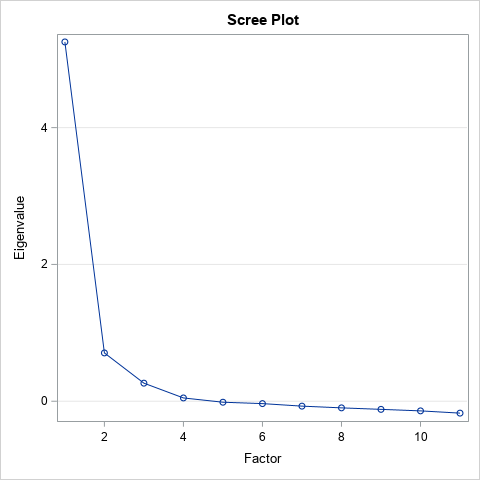


**Figure S3. Scree plot for number of factors selection, maximum likelihood extraction**

**Table S1a. Rotated factor pattern loadings**^1^

| Items | Factor 1 | Factor 2 | Factor 3 | Communality |
| --- | --- | --- | --- | --- |
|  | **Communication** | **Well-being** | **Engagement** |  |
| I would recommend UUH | -0.01498 | -0.1235 | **0.954174** | 0.739621 |
| I see myself working at UUH | 0.070953 | -0.03774 | **0.637249** | 0.445294 |
| I am motivated to do best | -0.01571 | 0.161756 | 0.371236 | 0.236765 |
| I have adequate opportunities | 0.516697 | 0.063172 | 0.205094 | 0.537581 |
| My supervisor keeps me informed | **0.874164** | 0.010132 | -0.08973 | 0.660659 |
| I can express opinions | **0.790231** | -0.01798 | 0.061172 | 0.683792 |
| My input is sought | **0.894614** | -0.03303 | 0.012093 | 0.779952 |
| I have access to tools | 0.203228 | 0.233948 | 0.364895 | 0.525763 |
| I have control over workload | 0.018529 | **0.803244** | -0.0703 | 0.587746 |
| My worked-related stress is manageable | 0.040454 | **0.738357** | 0.06233 | 0.657885 |
| Burnout is not a problem for me | -0.05017 | **0.872949** | -0.0525 | 0.650556 |
| ^1^ Maximum likelihood extraction with promax rotation | | | |  |

**Table S1b. Inter-factor correlations**^1^

| Items | Factor 1 | Factor 2 | Factor 3 |
| --- | --- | --- | --- |
| Factor 1 | 1.00 | - | - |
| Factor 2 | 0.64 | 1.00 | - |
| Factor 3 | 0.78 | 0.71 | 1.00 |
| ^1^ From maximum likelihood extraction with promax rotation | | | |


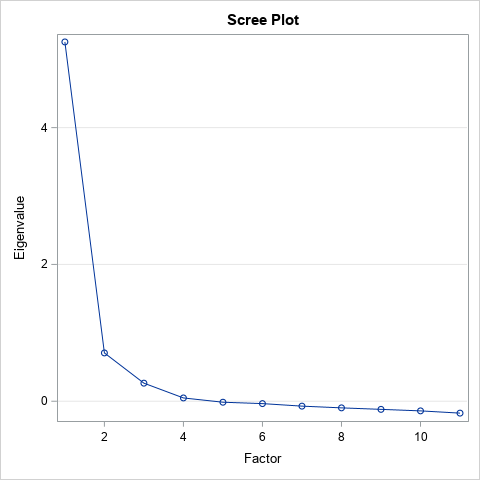


**Figure S4. Scree plot for number of factors selection, minimum residual extraction**

**Table S2a. Rotated factor pattern loadings**^1^

| Items | Factor 1 | Factor 2 | Factor 3 | Communality |
| --- | --- | --- | --- | --- |
|  | **Communication** | **Well-being** | **Engagement** |  |
| I would recommend UUH | 0.029997 | -0.10056 | **0.876799** | 0.692599 |
| I see myself working at UUH | 0.045838 | -0.05726 | **0.684475** | 0.463909 |
| I am motivated to do best | -0.04009 | 0.137535 | 0.424443 | 0.249545 |
| I have adequate opportunities | 0.484972 | 0.053034 | 0.254726 | 0.545086 |
| My supervisor keeps me informed | **0.87381** | 0.005251 | -0.07752 | 0.670189 |
| I can express opinions | **0.783787** | 0.00043 | 0.04732 | 0.674289 |
| My input is sought | **0.885698** | -0.02745 | 0.018537 | 0.779543 |
| I have access to tools | 0.199488 | 0.232133 | 0.370004 | 0.523738 |
| I have control over workload | 0.021419 | **0.78742** | -0.05289 | 0.584222 |
| My worked-related stress is manageable | 0.048559 | **0.743116** | 0.050025 | 0.658604 |
| Burnout is not a problem for me | -0.0523 | **0.863488** | -0.03695 | 0.650893 |
| ^1^ Minimum residual extraction with promax rotation | | | |  |

**Table 2b. Inter-factor correlations**^1^

| Items | Factor 1 | Factor 2 | Factor 3 |
| --- | --- | --- | --- |
| Factor 1 | 1.00 | - | - |
| Factor 2 | 0.63 | 1.00 | - |
| Factor 3 | 0.77 | 0.70 | 1.00 |
| ^1^ From minimum residual extraction with promax rotation | | | |

**Table S3. Associations with weighted factor scores^1^ for communication, well-being, and engagement (multivariate linear regression^2^)**

| Factor scores^1^ | Communication | Well-being | Engagement | |
| --- | --- | --- | --- | --- |
|  | $\hat{\beta}$_ADJ_ ^3^ (p-value) | $\hat{\beta}$_ADJ_ ^3^ (p-value) | $\hat{\beta}$_ADJ_ ^3^ (p-value) | |
| Age |  |  |  | |
| 30-39 | 0 [Reference] | 0 [Reference] | 0 [Reference] | |
| 40-49 | -0.15 (0.09) | **-0.18 (0.04)** | -0.13 (0.13) | |
| 50-59 | -0.20 (0.07) | -0.10 (0.35) | -0.11 (0.28) | |
| 60+ | -0.15 (0.21) | 0.19 (0.12) | -0.10 (0.41) | |
| Prefer not to answer | **-0.68 (0.00)** | **-0.55 (0.02)** | **-0.67 (0.01)** | |
| Everyone else | 0.08 (0.73) | 0.11 (0.64) | 0.25 (0.31) | |
|  |  |  |  | |
| Gender |  |  |  | |
| Female | 0 [Reference] | 0 [Reference] | 0 [Reference] | |
| Male | 0.09 (0.21) | **0.27 (0.00)** | 0.08 (0.28) | |
|  |  |  |  | |
|  |  |  |  | |
| Race Ethnicity |  |  |  | |
| White | 0 [Reference] | 0 [Reference] | 0 [Reference] | |
| Asian | -0.11 (0.44) | 0.09 (0.52) | -0.16 (0.26) | |
| Hispanic | -0.26 (0.32) | -0.08 (0.75) | -0.28 (0.29) | |
| Other | -0.36 (0.19) | 0.10 (0.72) | -0.10 (0.72) | |
| Prefer not to say | **-0.41 (0.00)** | -0.25 (0.06) | **-0.41 (0.00)** | |
|  |  |  |  | |
| Appointment Type |  |  |  | |
| Mostly clinical | 0 [Reference] | 0 [Reference] | 0 [Reference] | |
| Mostly other | **0.53 (0.00)** | 0.29 (0.09) | 0.33 (0.06) | |
| Mostly research | 0.08 (0.58) | 0.14 (0.37) | 0.03 (0.83) | |
|  |  |  |  | |
| Degree |  |  |  | |
| APC | 0 [Reference] | 0 [Reference] | 0 [Reference] | |
| MD | **0.25 (0.01)** | 0.16 (0.11) | 0.15 (0.12) | |
| ^1^ Weighted factor score created for domains of interest from iterated principal axis factor extraction EFA with promax rotation;  ^2^ All three domains (weighted factor scores) fit as outcomes simultaneously, Pillai test p-values for inclusion of predictors in multivariate model: age=0.01, gender=0.001, race/ethnicity=0.067, research/clinical=0.09, degree=0.07);  ^3^ Average change in domain with each one-unit increase in predictor, while holding all other predictors constant;  Bold = statistically significant (i.e. p < 0.05). Gray = on the boundary of statistical significance (i.e. 0.05 <p<0.10) | | | |  |


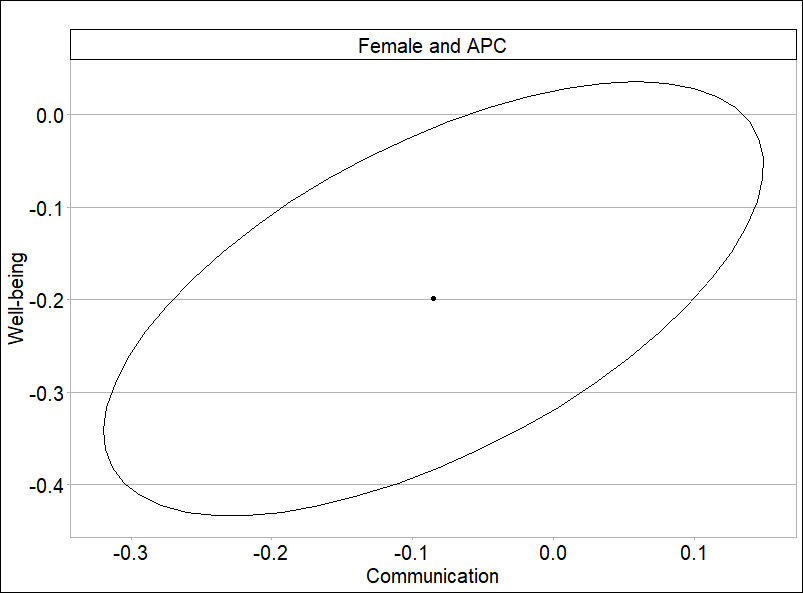

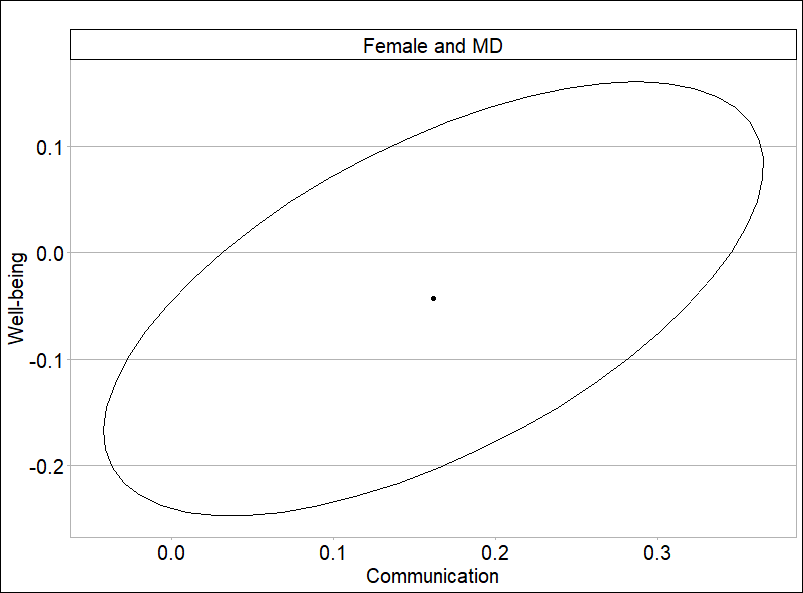


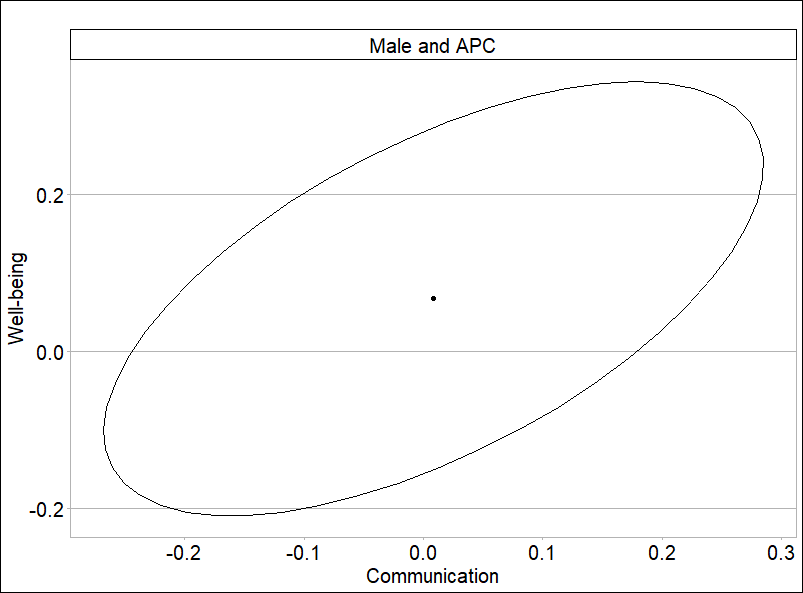

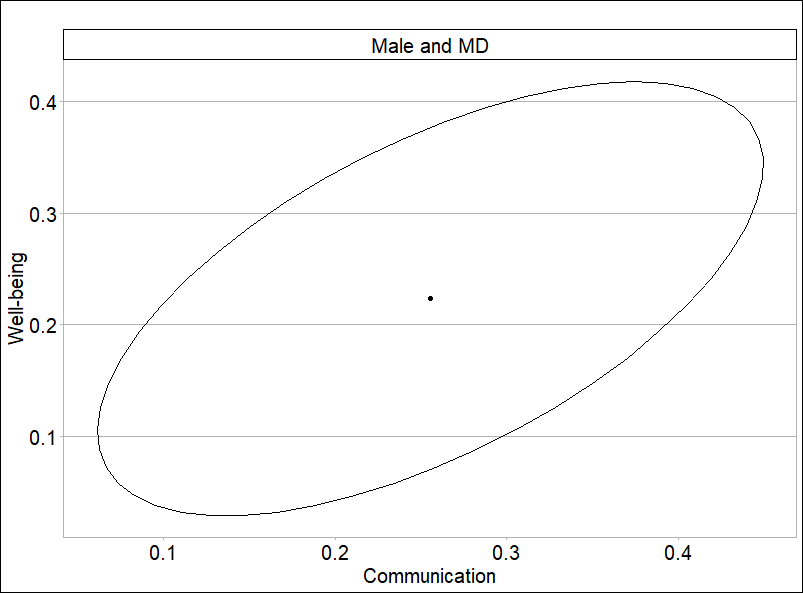


**Figure S5a: 95% confidence ellipse for communication and well-being (by gender and degree) while holding constant at age=30-39, race/ethnicity=white, and research/clinical=mostly clinical**


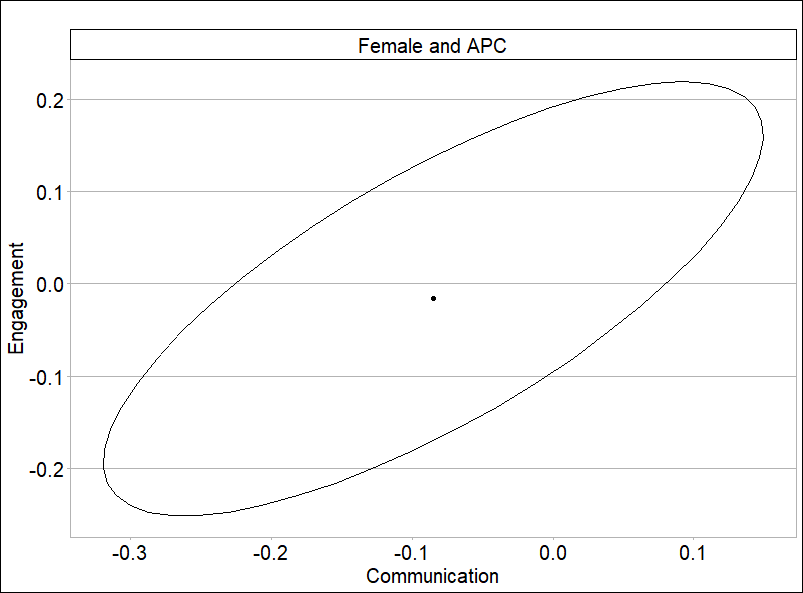

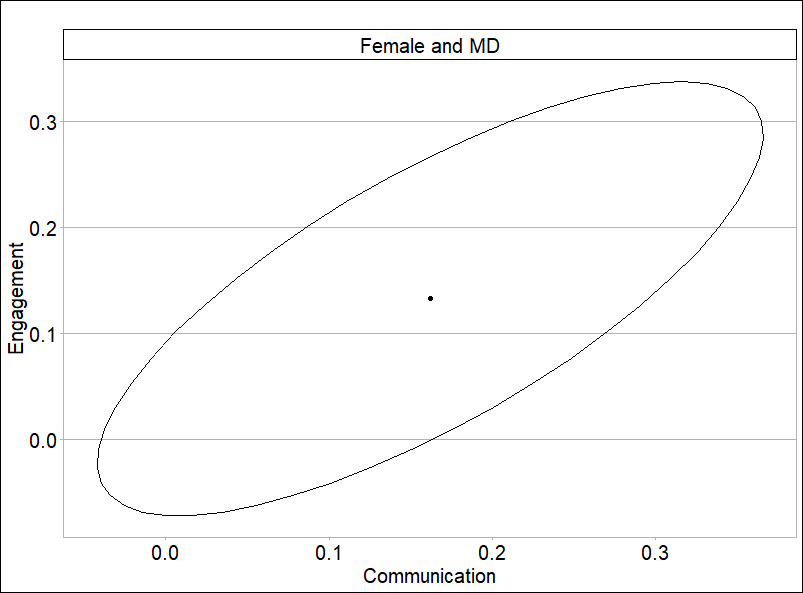


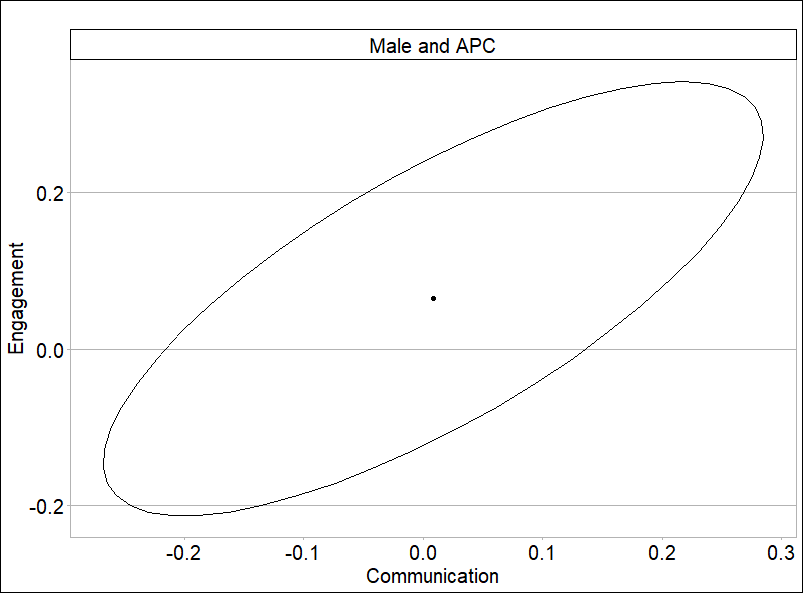

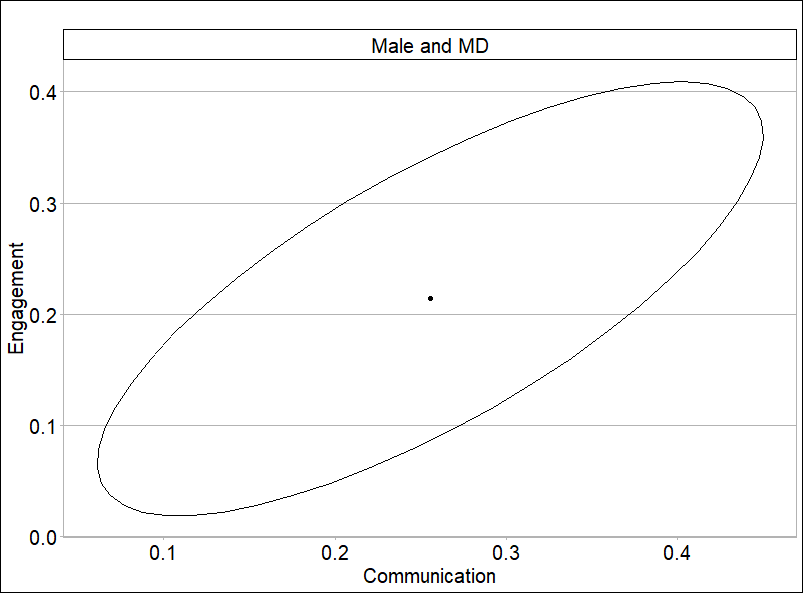


**Figure S5b: 95% confidence ellipse for communication and engagement (by gender and degree) while holding constant at age=30-39, race/ethnicity=white, and research/clinical=mostly clinical**


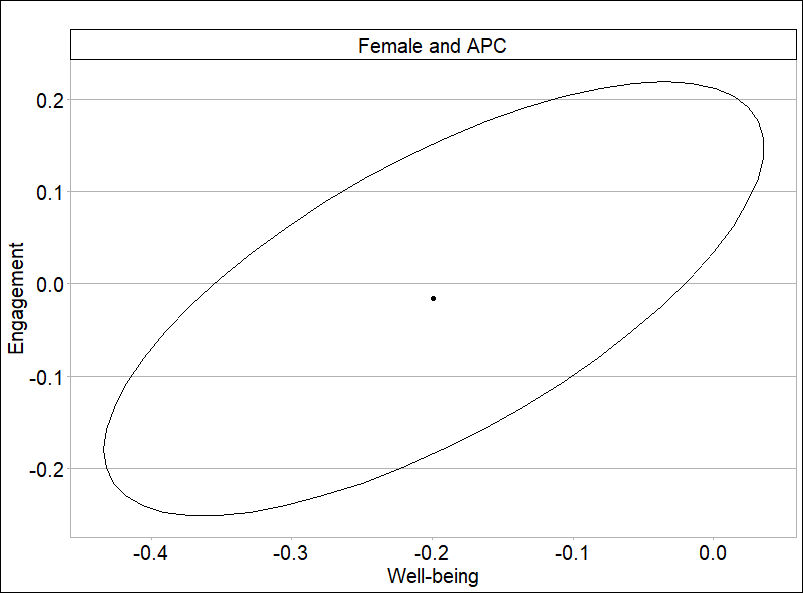

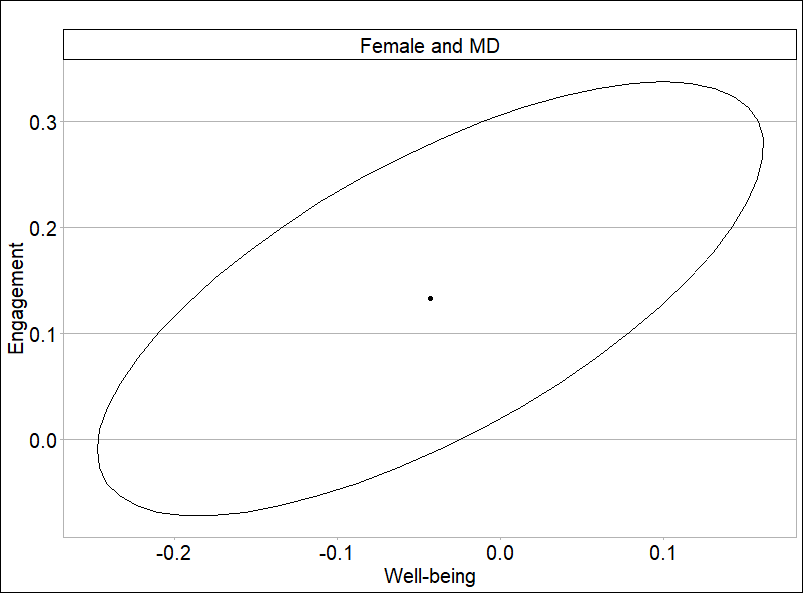


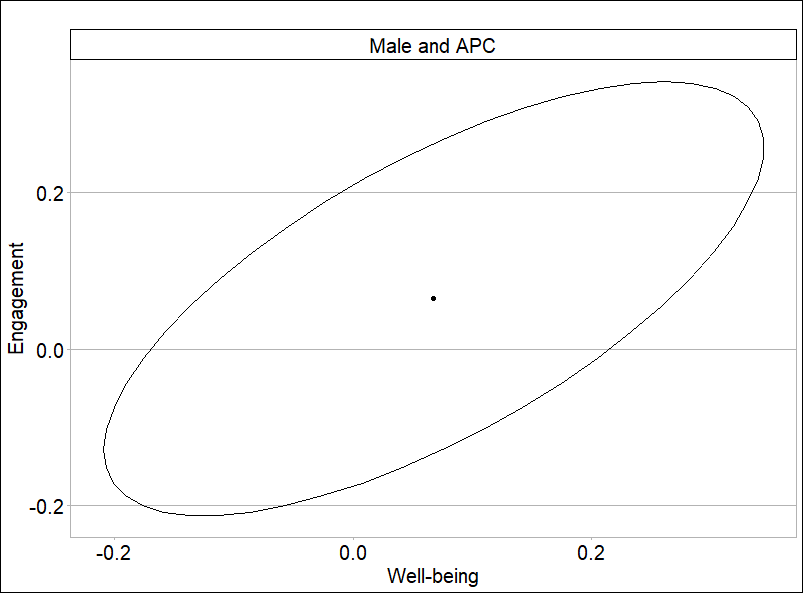

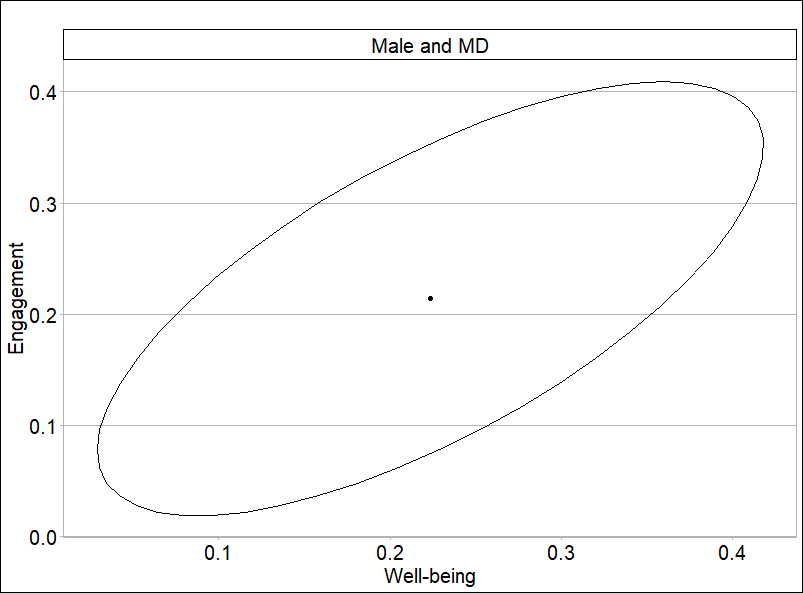


**Figure S5c: 95% confidence ellipse for well-being and engagement (by gender and degree) while holding constant at age=30-39, race/ethnicity=white, and research/clinical=mostly clinical**
